# Supplementary material for: A Platform Incorporating Trimeric Antigens into Self-Assembling Nanoparticles Reveals SARS-CoV-2-Spike Nanoparticles to Elicit Substantially Higher Neutralizing Responses than Spike Alone
Source: bioRxiv. 2020 Aug 22:2020.06.11.147496. Originally published 2020 Jun 12. Preprint. [Version 2] doi: 10.1101/2020.06.11.147496 (PMC7359518; doi:10.1101/2020.06.11.147496)
Supplement: Supplement 1 [file media-1.pdf]

Supplementary Table S1. Amino acid sequences of constructs for protein expression.

Construct name	Amino acid sequence
LuS-N71-SpyTag	MDSKGSSQKGSRLLLLLVVSNLLLPQGVVGAHIVMVDAYKPTKGSGSAMQIYEGKLTAEGLRFGIVASRFNHALVDRL<br>VEGAIDAIVRHGGREEDITLVRVPGSWEIPVAAGELARKENISAVIAIGVLIRGATPHFDYIASEVSKGLADLSLELRKPIT<br>FGVITADTLEQAIERAGTKHGNKGWEAALSAIEMANLFKSLRGGLVPRGSHHHHHHSAWSHPQFEK
Ferritin-N96-SpyTag	MDSKGSSQKGSRLLLLLVVSNLLLPQGVVGQHHHHHHHHSAWSHPQFEKGGLVPRGGAHIVMVDAYKPTKGGGSG<br>DPMLSKDIIKLLNEQVNKEMQSSNLYMSMSSWCYTHSLDGAGLFLFDHAAEEYEHAKKLIIFLNENNVPVQLTSISAPE<br>HKFEGLTQIFQKAYEHEQNISESINNIVDHAIKSKDHATFNFLQWYVAEQHEEEVLFKDILDKIELIGNENHGLYLADQY<br>VKGIAKSRKS
RSV F-SpyCatcher	MELLILKANAITTILTAVTFCFASGQNITEEFYQSTCSAVSKGYLGALRTGWYTSVITIELSNIKEIKCNGTDAKVKLIKQE<br>LDKYKNAVTDLQLLMQSTPATGSGSAIASGVAVCKVLHLEGEVNKIKSALLSTNKAVVSLSGCGVSVLTFKVLDLKNYI<br>DKQLLPILNKQSCSIPNIETVIEFQQKNNRLLEITREFSVNAGVTTPVSTYMLTNSELLSLINDMPITNDQKKLMSNNVQI<br>VRQQSYSIMCIIKEEVLAYVVQLPLYGVIDTPCWKLHTSPLCTTNTKEGSNICLTRTDRGWYCDNAGSVSFFPQAETC<br>KVQSNRVFCDTMNSRTLPSEVNLCNVDIFNPKYDCKIMTSKTDVSSSVITSLGAIVSCYGKTKCTASNKCRGIIKTFSN<br>GCDYVSNKGVDTVSVGNTLYYVNKQEGQSLYVKGEPIINFYDPLVFPSDEFDASISQVNEKINQSLAFIRKSDELLSAIG<br>GYIPEAPRDGQAYVRKDGEWVLLSTFLGSGDSATHIKFSKRDEDGKELAGATMELRDSSGKTISTWISDGQVKDFYL<br>YPGKYTFVETAAPDGYEVATAITFTVNEQGQVTVNGKATKGDAHIGSGLVPRGSHHHHHHSAWSHPQFEK
PIV3 F-SpyCatcher	MYSMQLASCVTLTLVLLVNSQIDITKLQHVGVLVNSPKGMKISQNFETRYLILSLIPKIEDSNSCGDQQIKQYKRLLDRLII<br>PLYDGLKLQKDVIVTNQESNENTDPRTERFFGGVIGTIALGVATSAQITAAVALVEAKQAKSDIEKLKEAIRDTNKAVQS<br>VCSSVGNCIVAIKSVQDYVNKEIVPSIARLGCEAAGLQLGIALTQHYSELTNCFGDNIGSLQEKGIKLQCIASLYRTNITEI<br>FTTSTVDKYDIYDLLFTESIKVRVIDVDLNDYSITLQVRLPLLTRLLNTQIYKVDSISYNIQNREWYIPLPSHIMTKGAFLG<br>GADVKECIEAFSSYICPSDPGFVLNHEMESCLSGNISQCPRTTVTSDIVPRYAFVNGGVVANCITTTCTCNGIGNRINQ<br>PPDQGVKIITHKECNTIGINGMLFNTNKEGTLAFYTPDDITLNNSVALDPIDISIELNKVKSDLEESKEWYRRSNQKLSAI<br>EDKIEEILSKIYHIENEIARIKKLIGEAPGGSGGDSATHIKFSKRDEDGKELAGATMELRDSSGKTISTWISDGQVKDFYL<br>YPGKYTFVETAAPDGYEVATAITFTVNEQGQVTVNGKATKGDAHIGSGLVPRGSHHHHHHSAWSHPQFEK
SARS-CoV-2 spike-SpyCatcher*	MGWSCIILFLVATATGVHSAPELLGGPSVFLFPPKPKDTLMISRTPEVTCVVVDVSHEDPEVKFNWYVDGVEVHNAKT<br>KPREEQYNSTYRVVSVLTVLHQDWLNGKEYKCKVSNKALPAPIEKTISKAKGQPREPQVYTLPPSRDELTKNQVSLY<br>CLVKGFYPSDIAVEWESNGQPENNYKTTPPVLDSDGSFFLYSKLTVDKSRWQQGNVFSCSVMHEALHNHYTQKSLS<br>LSPGKGGGGSGGGGSGGGGSGGGGSAPELLGGPSVFLFPPKPKDTLMISRTPEVTCVVVDVSHEDPEVKFNWYVD<br>GVEVHNAKTKPREEQYNSTYRVVSVLTVLHQDWLNGKEYKCKVSNKALPAPIEKTISKAKGQPREPQVYTLPPSRDE<br>LTKNQVSLTCLVKGFYPSDIAVEWESNGQPENNYKTTPPVLDSDGSFFLTSKLTVDKSRWQQGNVFSCSVMHEALH<br>NHYTQKSLSLSPGKGGSGGGGSGGLEVLFQGPQCVNLTTRTQLPPAYTNSFTRGVYYPDKVFRSSVLHSTQDLFLP<br>FFSNVTWFHAIHVSGTNGTKRFDNPVLPFNDGVYFASTEKSNIIRGWIFGTTLDSKTQSLLIVNNATNVVIKVCEFQFC<br>NDPFLGVYYHKNNKSWMESEFRVYSSANNCTFEYVSQPFLMDLEGKQGNFKNLREFVFKNIDGYFKIYSKHTPINLV<br>RDLPQGFSALEPLVDLPIGINITRFQTLLALHRSYLTPGDSSSGWTAGAAAYYVGYLQPRTFLLKYNENGTITDAVDCA<br>LDPLSETKCTLKSFTVEKGIYQTSNFRVQPTESIVRFPNITNLCPFGEVFNATRFASVYAWNRKRISNCVADYSVLYNS<br>ASFSTFKCYGVSPTKLNDLCFTNVYADSFVIRGDEVRQIAPGQTGKIADYNYKLPDDFTGCVIAWNSNNLDSKVGGNY<br>NYLYRLFRKSNLKPFERDISTEIYQAGSTPCNGVEGFNCYFPLQSYGFQPTNGVGYQPYRVVVLSFELLHAPATVCGP<br>KKSTNLVKNKCVNFNFNGLTGTGVLTESNKKFLPFQQFGRDIADTTDAVRDPQTLEILDITPCSFGGVSVITPGTNTSN<br>QVAVLYQDVNCTEVPVAIHADQLTPTWRVYSTGSNVFQTRAGCLIGAEHVNNSYECDIPIGAGICASYQTQTNSPGSA<br>SSVASQSIIAYTMSLGAENSVAYSNNSIAIPTNFTISVTTEILPVSMTKTSVDCTMYICGDSTECSNLLLQYGSFCTQLNR<br>ALTGIAVEQDKNTQEVFAQVKQIYKTPPIKDFGGFNFSQILPDPSKPSKRSFIEDLLFNKVTLADAGFIKQYGDCLGDIA<br>ARDLICAQKFNGLTVLPPLLTDEMIAQYTSALLAGTITSGWTFGAGAALQIPFAMQMAYRFNGIGVTQNVLYENQKLIA<br>NQFNSAIGKIQDSLSSTASALGKLQDVVNQNAQALNTLVKQLSSNFGAISSVLNDILSRLDPPEAEVQIDRLITGRLQSL<br>QTYVTQQLIRAAEIRASANLAATKMSECVLGQSKRVDFCGKGYHLMSFPQSAPHGVVFLHVTYVPAQEKNFTTAPAIC<br>HDGKAHFPREGVFVSNGTHWFVTQRNFYEPQIITTDNTFVSGNCDVVIGIVNNTVYDPLQPELDSFKEELDKYFKNHT<br>SPDVDLGDISGINASVVNIQKEIDRLNEVAKNLNESLIDLQELGKYEQGSGYIPEAPRDGQAYVRKDGEWVLLSTFLGR<br>SGGGLVPQQSGDSATHIKFSKRDEDGKELAGATMELRDSSGKTISTWISDGQVKDFYLYPGKYTFVETAAPDGYEVA<br>TAITFTVNEQGQVTVNGKATKGDAHI

* This amino acid sequence includes a single chain Fc purification tag (see reference 38).
